# Ethnicity influences phenotype and clinical outcomes: Comparing a South American with a North American inflammatory bowel disease cohort

**DOI:** 10.1097/MD.0000000000030216

**Published:** 2022-09-09

**Authors:** Tamara Pérez-Jeldres, Benjamín Pizarro, Gabriel Ascui, Matías Orellana, Mauricio Cerda-Villablanca, Danilo Alvares, Andrés de la Vega, Macarena Cannistra, Bárbara Cornejo, Pablo Baéz, Verónica Silva, Elizabeth Arriagada, Jesús Rivera-Nieves, Ricardo Estela, Cristián Hernández-Rocha, Manuel Álvarez-Lobos, Felipe Tobar

**Affiliations:** a Department of Gastroenterology, Faculty of Medicine, Pontifical Catholic University of Chile, Santiago, Chile; b Instituto Chileno-Japonés, University of Chile, Santiago, Chile; c Radiology Department, Hospital Clínico Universidad de Chile, Santiago, Chile; d La Jolla Institute for Allergy and Immunology, San Diego, CA; e Department of Computer Science, Faculty of Physical Sciences and Mathematics of the University of Chile, Santiago, Chile; f Integrative Biology Program, Institute of Biomedical Sciences, Center for Medical Informatics and Telemedicine, Faculty of Medicine, Universidad de Chile, Santiago, Chile; g Department of Statistics, Pontifical Catholic University of Chile, Santiago, Chile; h Inflammatory Bowel Disease Center, Division of Gastroenterology, University of California, San Diego, La Jolla, CA; i Initiative for Data & Artificial Intelligence, University of Chile; j Center for Mathematical Modeling, University of Chile, Santiago, Chile.

**Keywords:** ethnicity, IBD, machine learning

## Abstract

Inflammatory bowel disease (IBD), including ulcerative colitis (UC) and Crohn disease (CD), has emerged as a global disease with an increasing incidence in developing and newly industrialized regions such as South America. This global rise offers the opportunity to explore the differences and similarities in disease presentation and outcomes across different genetic backgrounds and geographic locations. Our study includes 265 IBD patients. We performed an exploratory analysis of the databases of Chilean and North American IBD patients to compare the clinical phenotypes between the cohorts. We employed an unsupervised machine-learning approach using principal component analysis, uniform manifold approximation, and projection, among others, for each disease. Finally, we predicted the cohort (North American vs Chilean) using a random forest. Several unsupervised machine learning methods have separated the 2 main groups, supporting the differences between North American and Chilean patients with each disease. The variables that explained the loadings of the clinical metadata on the principal components were related to the therapies and disease extension/location at diagnosis. Our random forest models were trained for cohort classification based on clinical characteristics, obtaining high accuracy (0.86 = UC; 0.79 = CD). Similarly, variables related to therapy and disease extension/location had a high Gini index. Similarly, univariate analysis showed a later CD age at diagnosis in Chilean IBD patients (37 vs 24; *P* = .005). Our study suggests a clinical difference between North American and Chilean IBD patients: later CD age at diagnosis with a predominantly less aggressive phenotype (39% vs 54% B1) and more limited disease, despite fewer biological therapies being used in Chile for both diseases.

## 1. Introduction

Inflammatory bowel diseases (IBD) are chronic immunological disorders that compromise the gastrointestinal tract.^[1]^ IBD includes ulcerative colitis (UC) and Crohn disease (CD).^[1]^ Both conditions have been considered to affect individuals of European ancestry.^[2]^ Recently, changes have been observed in IBD epidemiology with the disorder emerging globally in people of different ethnicity and geographic regions with an increasing incidence in developing and newly industrialized countries.^[3]^ The rising IBD incidence in South America offers the opportunity to explore differences and similarities in disease presentation and outcomes across different geographic regions and ethnic groups.^[2]^ A better understanding of the epidemiology and IBD progression in different communities and ethnic groups is crucial to evaluate allelic effects in disease-related outcomes.^[4]^ In particular, an improved patient stratification that considers differences among patients of different ethnicities could lead to better medical approaches with precision medicine.^[4]^ Precision medicines aim in IBD is to utilize each patient’s specific clinical and biological characteristics to predict the disease course and tailor treatment to deliver optimal care.^[5]^

In the last years, artificial intelligence (AI) has gained relevance to favor a precision medicine approach for many diseases including IBD.^[6,7]^ In particular, AI and its data-processing techniques, based on machine learning (ML) and deep learning, introduce the efficient integration and interpretation of big datasets, leading to clinically translatable knowledge.^[8]^ ML is also starting to improve health care by unlocking the potential of large numbers of patients and biomedical datasets.^[6,8,9]^ For example, ML-based methods may be used to predict disease outcomes, patient stratification, disease progression, and therapy responses favoring personalized and precise medicine with positive impacts on health expenditures, health care, and safety.^[8]^ ML stands as a promising resource to handle the enormity and complexity of biomedical data, where manual analysis is both untenable and inefficient.^[8]^ Moreover, many diseases including IBD involve a complex constellation of dynamic changes that vary in each patient.^[7,8]^ In this context, ML can assist medical and biomedical scientists by identifying and summarizing meaningful patterns from large datasets.^[8]^

We applied an ML strategy to evaluate the differences in clinical phenotypes between the datasets of North American and Chilean patients with IBD. This strategy consisted of univariate analysis, followed by an unsupervised approach, with subsequent corroboration of the findings using supervised ML. The unsupervised approach shows the distribution of patients for each disease and separates them into 2 main groups according to cohort (Chilean vs North American) with a slight overlap. A supervised model was then trained to identify the cohort to correctly cluster patients into their respective groups with at least 80% accuracy.

## 2. Materials and Methods

### 2.1. Study groups

This is an observational study that includes 2 cohorts.

### 2.2. North American IBD cohort

This study enrolled IBD patients who underwent colonoscopy requested by their IBD physician and agreed to participate in the study (VA San Diego Healthcare System and University California San Diego [UCSD] Medical Center). The patients were enrolled in the study between 2016 and 2018. An IBD specialist evaluated each patient to define their disease subtype (UC or CD), location, and phenotype. The clinical data were collected by an IBD physician. All patients were born in the US, and this cohort corresponds to an IBD group of patients who attended 1 of the leading Southern California IBD centers.

### 2.3. Chilean IBD cohort

We conducted a retrospective medical record review of adult IBD patients attending 2 Chilean IBD clinics (the Pontifical Catholic University of Chile and San Borja Arriarán Hospital). All patients were scheduled for colonoscopy between 2016 and 2019 as requested by their doctors.

### 2.4. Sample size

The sample size was calculated by considering the distribution of the mean age at the diagnosis of CD and UC. Damas et al^[10]^ reported a mean age at diagnosis of 27.0 ± 15.2 years for the non-Hispanic white group, and 38.0 ± 15.5 years Hispanics. A 2-sample Satterhwaite *t* test, assuming unequal variances, was applied. A minimum of 42 patients were required in each group/country (total number of patients = 84; alpha = 0.05; power = 90%). The final sample size was 265 (Chilean cohort, 177; North American cohort, 88).

### 2.5. Data analysis

#### 2.5.1. Univariate analysis.

An exploratory univariate analysis of demographic, clinical, and laboratory characteristics was performed using the database of patients with UC and CD from the Chilean and North American cohorts. Continuous variables were evaluated using *t* tests or Mann–Whitney *U* tests, as appropriate. Categorical variables were assessed the χ^2^ tests or Fisher exact test as appropriate. Data were analyzed using GraphPad Prism version 9.1.2 (GraphPad Software, San Diego, CA). The significance of the differences in quantitative variables was evaluated using the Mann–Whitney *U* test for 2 independent groups. Categorical variables were evaluated using Fisher exact test. Statistical significance was set at a *P* value of < .05. For plotting, we used the seaborn and matplotlib Python libraries.

#### 2.5.2. Unsupervised and supervised analysis.

Unsupervised analysis aims to identify similar observations across patients and to group them into clusters. Unsupervised learning methods operate on unlabeled data; thus, their aim is to discover common patterns in the training dataset.^[11]^ The metadata included the disease phenotype, current and prior treatments, disease activity and location, complications, and relevant covariates. To reduce the dimensionality of the IBD clinical data, we performed PCA, kernel PCA, t-distributed stochastic neighbor embedding, uniform manifold approximation, and projection (UMAP), and multidimensional scaling. Two supervised clustering algorithms were used (hierarchical agglomerative clustering and k-means). In addition, we performed a statistical analysis between the identified clusters: a 1-way analysis of variance for continuous clinical variables and *x*^2^ test for binary variables to determine which clinical characteristics were different between these clusters.

To determine which variables better defined the cohorts of North American and Chilean patients with IBD, a supervised ML method was used. Recall that supervised learning methods predict an outcome (dependent variable) based on a set of features (independent variable),^[12]^ and thus, they operate over *labeled* datasets.^[12]^ Supervised ML algorithms can be divided into 2 categories: classification and regression.^[12]^ Classification algorithms predict categorical outcomes (e.g., high, or low), whereas regression algorithms predict continuous outcomes (e.g., blood pressure, weight).^[12]^ Within the supervised classification methods, we used a Random Forest approach to predict relevant clinical variables that differentiate 2 populations. This method is computationally inexpensive and performs both regression and classification tasks, particularly in situations in which there are fewer observations than variables.^[13]^

To develop the model, we divided the data into 2 groups: one for training and another for testing the model. This strategy ensures better generalization of the model. After running several experiments, test sizes ranging from 10% to 99%, 80%, and 20% of the data were used for training and testing. The software randomly separated the dataset into 2 groups without any external supervision and stratified by class to reduce class imbalance.

A grid search 5-Fold Cross validation scheme was used to train and adjust the models. The random forest (RF) parameters explored were the number of trees, maximum depth, minimum sample split, and the minimum number of sample leaves. The Gini index was used as the split criterion. The model with the best cross-validation performance was selected.

For RF the final model hyperparameters were a max depth of features UC = 15, CD = 5; max features UC= “log2,” CD= “auto”; trees UC = 50, CD = 59; min sample leaf UC = 1, CD = 1; min sample split UC = 1, CD = 1.

Accuracy, precision, recall, F1-score, macro average, and weighted average accuracy metrics of the models were obtained.^[14]^ Accuracy is the proportion of the total number of correct predictions. Precision is the number of correctly identified class members divided by the number of times the model predicted that class. Recall is the number of class members that the classifier identified correctly, and divided by the total number of members in that class. The F1-score combines recall and precision, and identifies whether the classifier is good at identifying class members.^[14]^

To further confirm the RF results, we compared the results of several supervised ML models without tuning (super vector machine, logistic regression, AdaBoost, multilayer perceptron (MLP), and linear discriminant analysis).^[12,15–18]^ The data were split into 2 groups, 80% for training and 20% for testing, as in our RF. Additionally, we developed a generalized linear model to predict the cohort (Chilean or North American) using R (version 4.1.1; 2021-08-10, RStudio, Boston, MA) and *dplyr, mlogit, tidyr* libraries. Variables with more than 30% of missing values were excluded. Mean imputation was used for the remaining variables.

For RF, the predictive importance of each clinical variable in the final model was estimated by using the Gini index. A higher Gini index value indicated a more relevant variable in the model. All the mentioned analyses were performed using Python version 3.8 (Python Software Foundation, Beaverton, OR) and the *sklearn* librar*y*.

### 2.6. Ethical considerations

This study used a dataset of patients diagnosed with CD or UC managed by the UCSD, San Diego, CA, USA (VA San Diego Healthcare System and UC San Diego Medical Center). This dataset was collected under the Institutional Review Board of the UCSD approved protocol numbers H130266 and 161756. Chilean patient data were collected with the approval of the ethical boards of both the institutions. The Institutional Review Board approved the protocol numbers 200609001 (Pontifical Catholic University of Chile, Santiago, Chile) and 72/623 (Hospital San Borja Arriarán).

## 3. Results

### 3.1. Univariate analysis

This study included 265 patients diagnosed with UC (n = 173) and CD (n = 92; Tables 1 and 2). The North American cohort included 88 patients (UC = 65; CD = 23) and the Chilean cohort included 177 patients (UC = 108; CD = 69). Each cohort (North American and Chilean) was analyzed according to disease subtype, UC, or CD. For both subtypes, the proportion of women was significantly higher in the Chilean cohort (UC = 63% Chilean women vs 28% North American women, *P* ≤ .0001; CD = 58% Chilean women vs 30% North American women, *P* = .01). Disease duration was lower in the Chilean cohort than in the North American cohort (median UC = 6 vs 10 years, *P* = .01; CD = 6 vs 16 years, *P* = .04), and the age at diagnosis was significantly lower in the North American vs Chilean CD group (median 24 vs 37 years, *P* = .005). Both cohorts showed significant differences in endoscopic activity, C-reactive protein, and albumin levels for each disease. The use of biological therapy was significantly higher in the North American group than that in the Chilean group.

**Table 1:** Demographic. Clinical disease characteristics and medication history of the patients in UC patients.

	North American	Chilean	*P* value
N	65 45 (69%) Caucasian/1 (1%) African American/18 (28%) Hispanic-Latin/1 (1%) Middle Eastern	108 104 (96%) Latin/4 (4%) Latin-Mapuche	
Age, yr,* median (max–min)	42 (19–88)	40 (16–77)	NS
Duration of Disease, yr, median (max–min)	10 (0–35)	6 (0–53)	.01
Age at diagnosis, yr, median (max–min)	28 (11–62)	31 (10–77)	NS
Sex	Female 18 (28%)/Male 47 (72%)	Female 68 (63%)/Male 40 (37%)	<.0001
Smoking (yes/no/no data)	1 (2%)/39 (60%)/25 (38%)	19 (18%)/88 (81%)/1 (1%)	.01
Surgery (yes/no)	4 (6%)/61 (94%)	7 (6%)/101 (94%)	NS
History of hospitalization (yes/no/no data)	22 (34%)/14 (22%)/29 (45%)	55 (51%)/53 (49%)	NS
Family history of IBD (yes/no/no data)	4 (6%)/44 (68%)/17 (26%)	8 (7%)/98 (91%)/2 (2%)	NS
Montreal UC			<.0001
Extensive colitis (E3)	37 (57%)	54 (50%)	
Left colitis (E2)	7 (11%)	27 (25%)	
Proctitis (E1)	0 (0%)	27 (25%)	
No data registered	21 (32%)	0	
White cell count ×10^6^/L (normal range 4000–11,000), mean (max–min)	7150 (3800–12,800)	6825 (3400–21,440)	NS
Hemoglobin (g/L)* (normal range 12–18), mean (max–min)	13.4 (8.5–16.7)	13.3 (7.1–16.9)	NS
Platelets, ×10^6^/L (normal range 150,000–450,000), mean (max–min)	269,000 (134,000–564,000)	285,500 (57,000–601,000)	NS
C reactive protein (mg/dL) normal range < 0.5	3 (0.03–8)	6 (0.03–15.88)	.01
Erythrocyte sedimentation rate (mm/h) normal range < 30	8 (2–28)	9 (1–86)	NS
Albumin (g/L) normal range (3.5–5.5)	4.3 (3–4.6)	4.5 (2.5–5.2)	.008
Total Mayo Score	1 (011)	2 (0–9)	NS
Endoscopic Mayo score 0/1/2/3/NA	25/10/2/2/22	32/25/38/13	.0002
Current therapies (yes/no/no data)			
Steroids	13 (20%)/52 (80%)	15 (14%)/93 (86%)	NS
Biological	30 (46%)/35 (%4%)	9 (8.3%)/99 (91.7%)	<.0001
Anti-TNF-α	18 (28%)/47 (72%)	9 (8.3%)/99 (91.7%)	.001
Immunomodulators	22 (34%)/43 (66%)	38 (35%)/69 (64%)/1 (1%)	NS
Aminosalicylates	16 (35%)/49 (65%)	84 (77%)/24 (22%)/1 (1%)	<.0001
History of therapies			
Steroids	47 (72%)/3 (5%)/15 (23%)	89 (82%)/13 (12%)/6 (6%)	NS
Immunomodulators	31 (48%)/14 (22%)/20 (30%)	53 (49%)/49 (45%)/6 (6%)	NS
Biological therapies	36 (55%)/9 (14%)/20 (31%)	18 (17%)/84 (83%)	<.0001
Naive anti-TNF-α	13 (20%)/52 (80%)	91 (84%)/17 (16%)	<.0001

IBD = inflammatory bowel disease, NS = not significant, TNF = tumor necrosis factor, UC = ulcerative colitis.

**Table 2:** Demographic, clinical disease characteristics, and medication history of the patients in CD patients.

	North-American	Chilean	*P* value
N	23 16 (70%) Caucasian/4 (17%) Hispanic-Latin/2 (9%) another Pacific Island/1 (4%) Asian	69 68 (99%) Latin/1 (1%) Latin-Mapuche	.5725
Age, yr, median (max–min)	45 (27–67)	44 (19–80)	NS
Duration of disease, yr, mean (max–min)	16 (1–44)	6 (0–26)	.04
Age at diagnosis, yr, mean (max–min)	24 (11–58)	37 (14–67)	.005
Sex	7 (30%) female/16 (70%) male	40 (58%) female/27 (39%) male	.01
Smoking (yes/no/no data)	4 (17%)/16 (70%)/3 (13%)	14 (20%)/51 (74%)/4 (6%)	NS
Surgery (yes/no)	9 (39%)/14 (61%)	16 (23%)/53 (77%)	NS
History of hospitalization (yes/no/no data)	16 (70%)/4 (17%)/3 (13%)	36 (52%)/31 (45%)/2 (3%)	.04
Family history of IBD (yes/no)	3 (13%)/20 (87%)	6 (8.7%)/63 (91.3%)	NS
Montreal Crohn disease			
A1/A2/A3/No data	2/15/2/4	4/40/25/0	.0008
Ileal (L1)	2 (9%)	18 (26%)	.0002
Colonic (L2)	4 (17%)	35 (51%)	
Ileocolonic (L3)	17 (74%)	16 (23%)	
Upper compromise (L4)	2 (9%)	8 (11.6%)	
B1 (inflammatory)	9 (39%)	37 (54%)	NS
B2 (structuring)	11 (48%)	17 (25%)	
B3 (penetrating)	3 (13%)	15 (21%)	
Perianal disease (yes/no)	9 (39%)/14 (61%)	14 (20)/55 (80%)	NS
White cells ×10^6^/L (normal range 4000–11,000), median (max–min)	7450 (4800–12,700)	6750 (1640–15,450)	NS
Hemoglobin (g/L) (normal range 12–18), median (max–min)	12.35 (7.7–15.7)	12.65 (8.8–17.2)	NS
Platelets ×10^6^/L (normal range 150,000–450,000), mean (max–min)	271,500 (155,000–699,000)	292,000 (140,000–629,000)	NS
C reactive protein (mg/dL) normal range < 0.5	4 (0.03–1.6)	7 (0.05–8.1)	.01
Erythrocyte sedimentation rate (mm/h) normal range < 30	11 (2–71)	11 (2–47)	NS
Albumin (g/L) normal range (3.5–5.5)	4.1 (2.9–4.9)	4.4 (2.9–5.1)	.0006
Harvey-Bradshaw Index	2 (0–9)	3 (0–18)	NS
SES-CD	0 (0–19)	4 (0–21)	.001
Current therapies (yes/no)			
Steroids	2 (9%)/21 (91%)	15 (22%)/54 (78%)	NS
Biological	20 (87%)/3 (13%)	16 (23%)/53 (77%)	.0001
Inmunomodulators	13 (6%)/10 (94%)	24 (35%)/45 (65%)	NS
History of therapies (yes/no)			
Steroids	19 (83%)/4 (17%)	52 (75%)/17 (25%)	NS
Inmunomodulators	19 (83%)/4 (17%)	48 (70%)/21 s(30%)	NS
Naive anti-TNF	1 (4%)/22 (96%)	48 (70%)/21 (30%)	<.0001

CD = Crohn disease, IBD = inflammatory bowel disease, NS = not significant, SES-CD = simple endoscopic score for Crohn’s disease.

When comparing North American and Chilean patients with IBD for each disease, significant differences were observed in the extension (UC, *P* < .0001) and location (CD, *P* < .0002) of the disease. Interestingly, a tendency toward a more limited disease extension was observed in the Chilean group. North American CD patients had a higher proportion of ileocolonic disease (L3/ileocolonic = 74% compared with L1/ileal = 9%, L2/colonic = 17%, and L4/upper digestive compromise = 9%), whereas in the Chilean population, colonic (L2 = 51%) and ileal (L1 = 26%) diseases were more frequent (Tables 1 and 2).

Because location (CD)/extension (UC) may have implications for the clinical approach, we evaluated the impact of continuous variables (age at diagnosis, disease duration, and laboratory parameters) on disease location (E1 = proctitis, E2 = left colitis, E3 = extensive colitis for UC, and L1 = ileal, L2 = colonic, L3 = ileocolonic, and L4 = upper digestive compromise for CD) in each group. Furthermore, we performed the same analysis for categorical variables (family history of IBD, sex, hospitalization for IBD, smoking habits, prior use of steroids, immunomodulators, biological therapy, history of discontinuation or failure of anti-TNF therapy, naive anti-TNF therapy, and current therapy with steroids, biological agents, anti-TNF, or immunomodulators). Tables S1 to S3 (Supplemental Digital Content, http://links.lww.com/MD/H81) shows the relationships between the clinical variables, extension (UC), and disease localization (CD). Albumin level was significantly related to extensive colitis/E3 in UC (*P* = .026), whereas in CD, it was related to the ileocolonic/L3 location of the disease (*P* = .005). Interestingly, despite fewer biological therapies in Chileans, more localized diseases are predominant.

#### 3.1.1. Dimensionality reduction.

**Figure 1.**
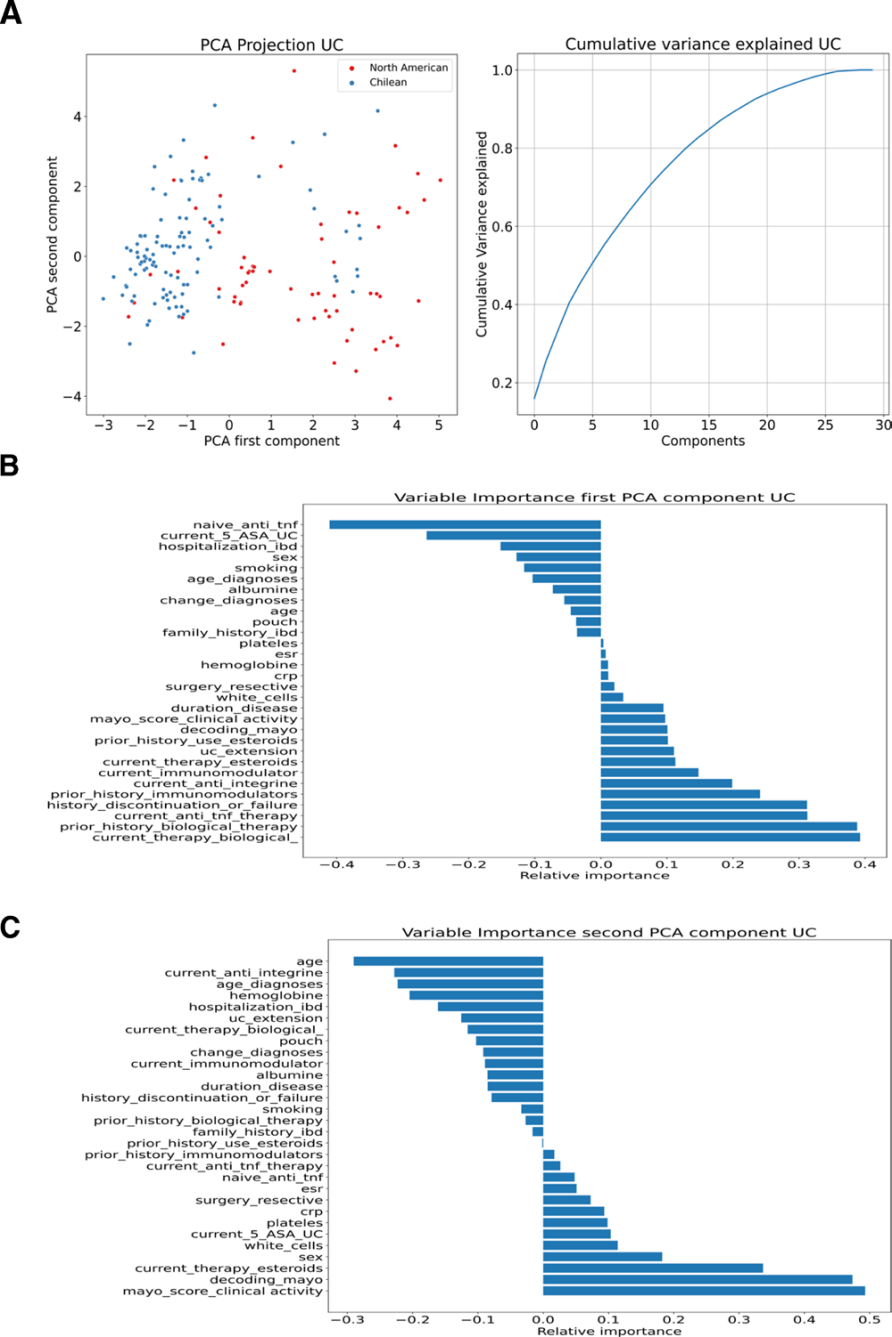
Analysis of clinical metadata of UC patients. To explore the relation between centers and clinical metadata (disease phenotype, treatments, disease activity, location disease, and complications), we used a PCA dimension reduction technique and then plotted the results in a 2D plot. These results show that 2 groups arise from the clinical data, one for Chilean patients and another for American patients. Also, PCA component variance explanation is shown. (A) PCA in UC. The patients were color-coded by cohort (Chilean blue dots and North American red dots). The first 5 PCA components represent the 50% cumulative variance. (B) Variable importance PC1. (C) Variable importance PC2. 2D = 2 dimensional, PC1 = first principal component, PCA = principal component analysis, UC = ulcerative colitis.

**Figure 2.**
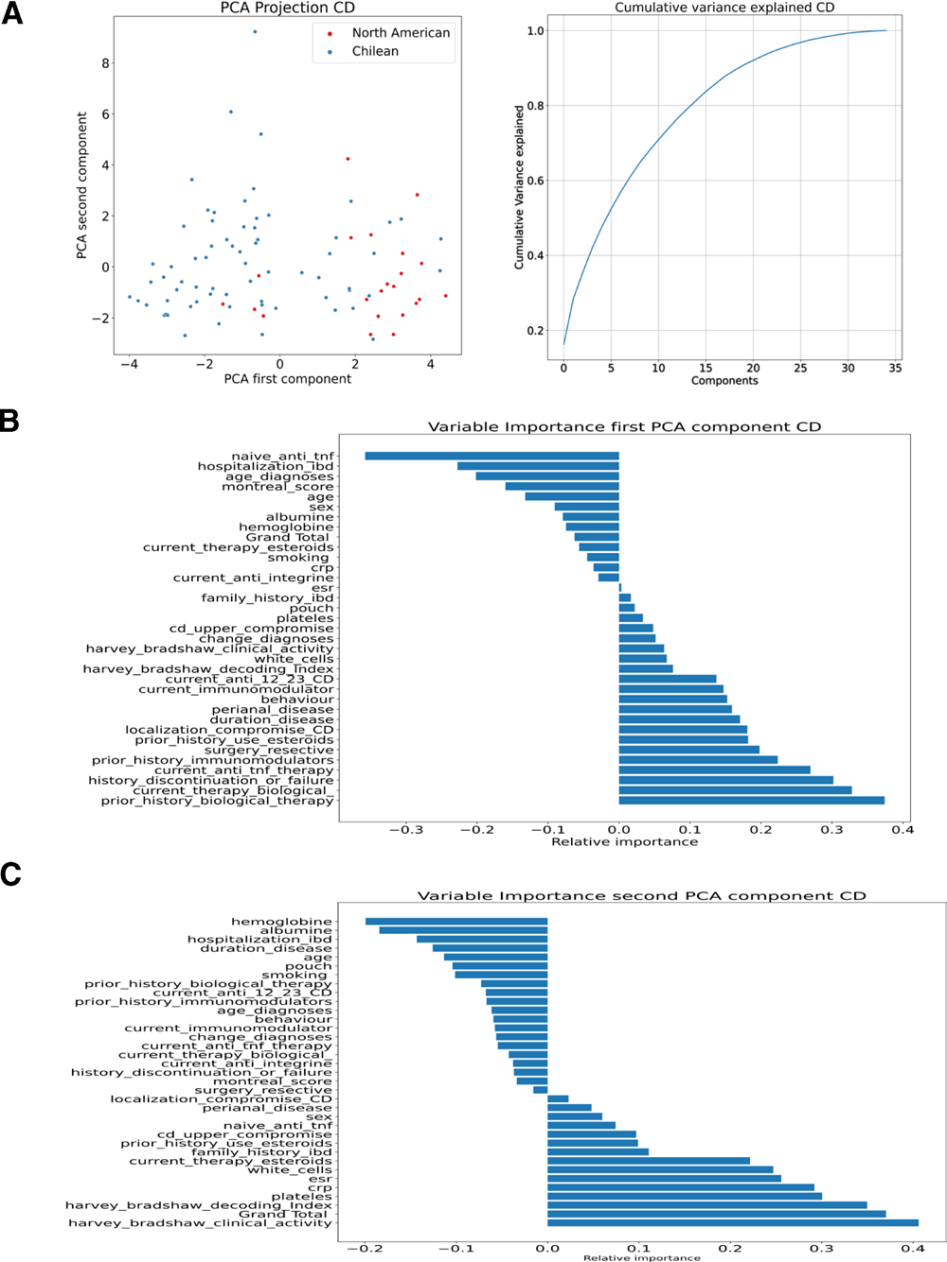
Analysis of clinical metadata of CD patients. Analysis of clinical metadata of CD patients. (A) PCA in CD. The patients are color-coded according to cohort: Chilean blue dots and North American red dots. The first 5 PCA components represent the 50% cumulative variance. (B) Variable importance PC1. (C) Variable importance PC2. * current_anti_12_23_CD = current use of ant IL-12 and IL-23; Grand_total = Total score SES-CD. CD = Crohn disease, IL = interleukin, PC1 = first principal component, PC2 = second principal component, PCA = principal component analysis, SES-CD = simple endoscopic score for Crohn’s disease, UC = ulcerative colitis.

Using PCA, 2 distinct groups were identified, supporting the differences between the North American and Chilean cohorts (Figs. 1A–C and 2A–C). The main clinical parameters contributing to first principal component (PC1) in UC were related to biological therapies, use of immunomodulators, use of steroids, and UC extension, and for second principal component (PC2), they were the Mayo score of clinical activity and steroid use. For CD, the main factors contributing to PC1 were related to the use of therapies (biological, immunomodulators, steroids), history of surgery, and localization of Crohn, whereas for PC2, the Harvey-Bradshaw Index of activity, endoscopic activity, platelet–white cell blood counts, C-reactive protein, and erythrocyte sedimentation rate.

**Figure 3.**
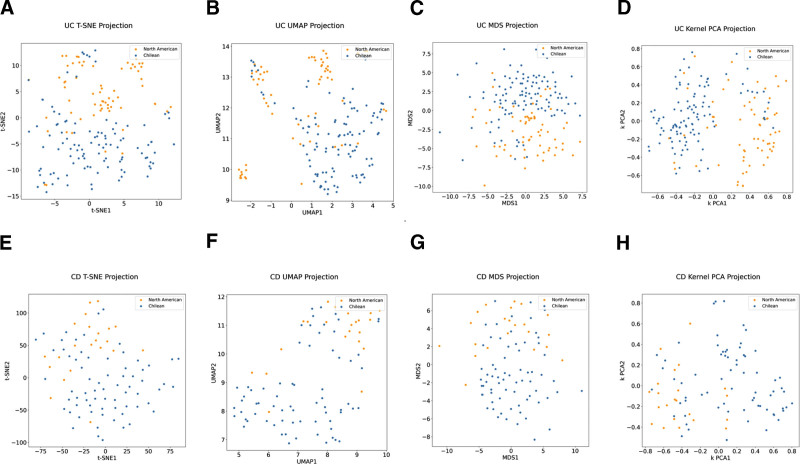
Dimensional reduction of IBD patient clinical data. (A–D) Ulcerative colitis patients’ data represented using t-SNE, UMAP, MDS, Kernel PCA (left to right). (E–H) Crohn Disease patients represented by the same algorithms. Patient Cohort or “center” is represented in blue for Chile and yellow for UCSD. nUC = 173, nCD = 92. IBD = inflammatory bowel disease, MDS = multidimensional scaling, PCA = principal component analysis, t-SNE = t-distributed stochastic neighbor embedding, UMAP = uniform manifold approximation, and projection, UCSD = University California San Diego.

In both diseases, current and past therapy history and location/extension were important in the PCA analysis to differentiate between the groups. However, PC1 and PC2 explained <30% of the variance in both diseases, indicating that PCA did not thoroughly explain the differences between the patients (Figs. 1A and 2A). Therefore, t-distributed stochastic neighbor embedding, UMAP, multidimensional scaling, and kernel PCA were implemented, which corroborated our results and separated the patients into 2 groups (mainly composed of North American and other Chilean populations) with some degree of overlap (Fig. 3). Therefore, we continued the analysis using these projections and 2 supervised clustering algorithms to investigate whether these projections represent patients with similar clinical characteristics. Because K-means clustering provided the best graphical representation (maximum separation) of the results obtained in the UMAP projections, we used these clusters for further analysis (see Figure S1, Supplemental Digital Content, http://links.lww.com/MD/H82, which illustrates the Clustering of IBD patients).

Interestingly, only clinical disease severity scores were significant between clusters after a multiple comparison post hoc Tukey test, while other variables were not (see Figure S2, Supplemental Digital Content, http://links.lww.com/MD/H83, which illustrates the clinical severity score for IBD patients). For patients with UC, the third cluster, comprising mainly of patients from the Chilean cohort, had a higher Mayo score than the other clusters. Similarly, for CD patients, the second cluster, also mainly from the Chilean cohort, had a higher Harvey-Bradshaw score than the other clusters. This difference was not observed by simply comparing the cohorts in univariate analysis (Tables 1 and 2). For binary clinical variables, therapies showed the most significant differences among the clusters (see Figure S3, Supplemental Digital Content, http://links.lww.com/MD/H84).

### 3.2. Supervised ML classifier

A supervised ML approach was used to train the RF. We developed this model to classify the patient countries for each cohort. In addition, using the Gini index from RF, we explored the most crucial variables for classifying North Americans and Chilean. In our UC model, we obtained a weighted accuracy of 0.86, with an f1 score of 0.84 for North American and 0.87 for Chilean IBD patients. For the CD model, we obtained a weighted accuracy of 0.80, with an f1 score of 0.71 for North American and 0.83 for Chilean patients, respectively (Table 3). Using RF models, 81% of North American and 89% of Chilean patients were correctly identified in the UC cohort and 83% and 77%, respectively, in the CD cohort, as shown in Figure S4 (Supplemental Digital Content, http://links.lww.com/MD/H85) which illustrates the confusion matrix for each classifier.

**Table 3:** Random Forest Classification report: (A) UC and (B) CD.

	Precision	Recall	f1-score	Support
(A) UC				
1 (North American)	0.87	0.81	0.84	16
2 (Chilean)	0.85	0.89	0.87	19
Accuracy			0.86	35
Macro average	0.86	0.85	0.86	35
Weighted average	0.86	0.86	0.86	35
(B) CD				
1 (North American)	0.62	0.83	0.71	6
2 (Chilean)	0.91	0.77	0.83	13
Accuracy			0.79	19
Macro average	0.77	0.80	0.77	19
Weighted average	0.82	0.79	0.80	19

CD = Crohn disease, UC = ulcerative colitis.

**Figure 4.**
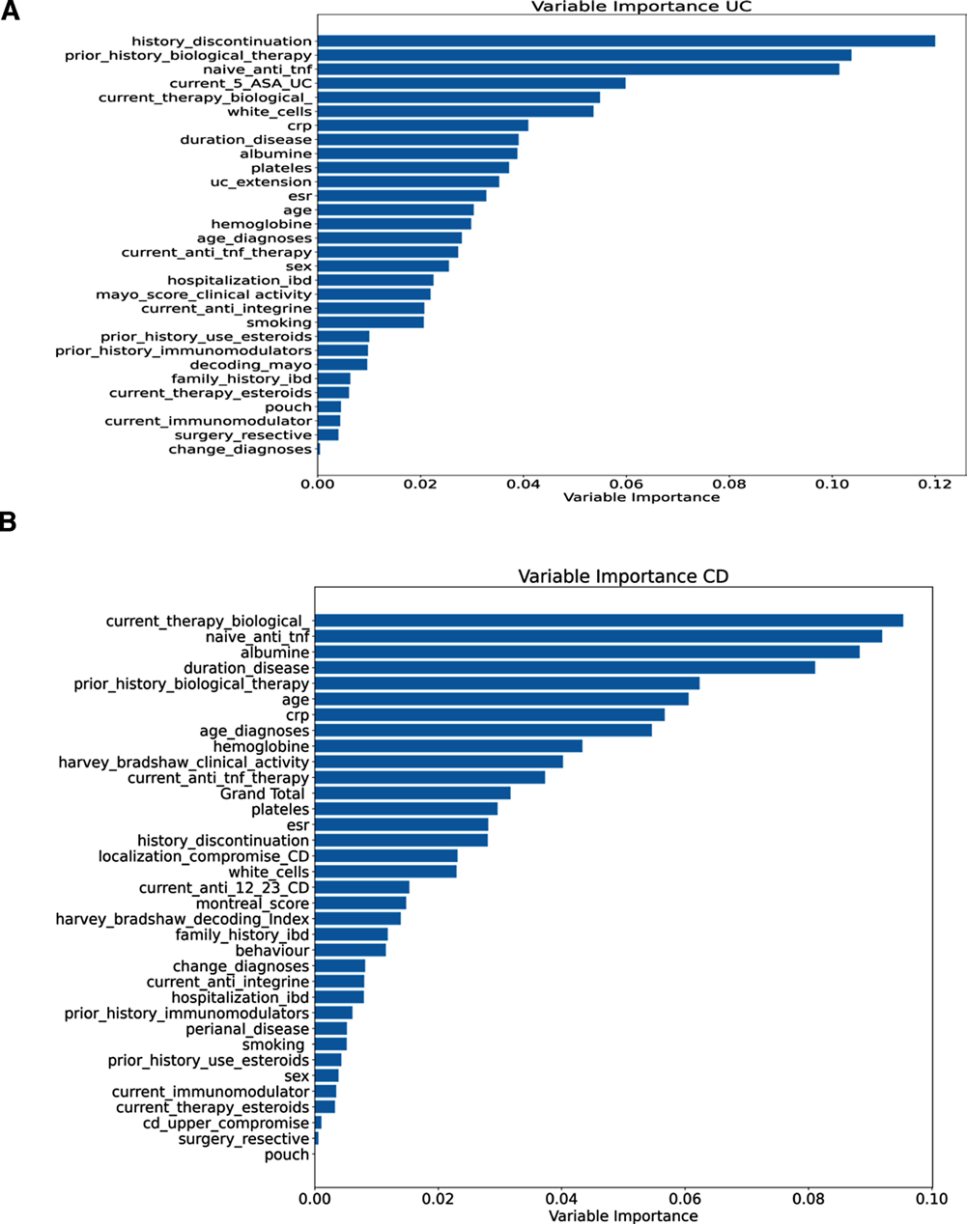
Variable importance for cohort prediction. To assess the importance of each variable for the random forest model, we plotted a horizontal bar graph, where the relative importance of each variable measured by the Gini index is shown. (A) the Gini index shows the main variables to predict the cohort (Chilean vs North American) in UC patients were related to use of biological therapies, aminosalicylates, inflammatory parameters, duration of disease, and UC extension. On the contrary, (B) shows that in CD patients the main variables were related to the use of therapies, duration of disease, inflammatory parameters, clinical activity score, and localization of the disease. *current_anti_12_23_CD = current use of ant IL-12 and IL-23; Grand_total = Total score SES-CD. CD = Crohn disease, IL = interleukin, SES-CD = simple endoscopic score for Crohn’s disease, UC = ulcerative colitis.

Using the Gini score index for each variable, we calculated the relative importance of each variable in predicting nationality (cohort; Fig. 4). According to the Gini index in the UC model, the main variables that allowed for the prediction of nationality were biological therapy use, amino salicylates, inflammatory parameters, disease duration, and UC extension. The main variables for CD were biological therapy, inflammatory parameters, disease duration, age, and CD location, supporting the results of the univariate and unsupervised methods.

In addition, we used a generalized linear model, which allowed us to model error distributions other than the normal distribution. We built various models for each disease to predict nationality (Chilean or North American) and incorporated variables with significant differences into the univariate analysis. Biological therapy was a significant predictor of nationality in both UC and CD patients (data not shown). Table S4 (Supplemental Digital Content, http://links.lww.com/MD/H86) compares the RF with different supervised ML algorithms (super vector machine, logistic regression, AdaBoost, MLP, and linear discriminant analysis), confirming our ability to predict a patient’s country using clinical data. Although all models performed well in the classification tasks in the UC cohort, RF and AdaBoost achieved the best performances. Similarly, RF and AdaBoost outperformed other methods in the CD cohort.

## 4. Discussion

Changes in the epidemiology of IBD have highlighted an increased incidence in South America, motivating the study of similarities and differences in clinical outcomes among patients attending California, who are mostly American Caucasians (North American cohort) and a Chilean population classified as Latino. These findings might help to untangle the phenotypic implications of genetic and environmental influences, offering important insights into disease pathogenesis. Moreover, translational studies that include IBD patients from diverse ethnicities in which IBD is still emerging are necessary, and tools such as ML can aid in the better stratification of patients with IBD. To analyze our data, we used an ML approach to validate our results in a complementary manner, which could support the robustness of our results. Future studies on datasets will increasingly incorporate more data science using ML and a deep-learning approach.

This aspect is crucial considering the heterogeneous clinical course of IBD, in which up to 20% of IBD patients require colectomy for refractory disease and more than half of the patients with CD require surgery within 10 years of diagnosis, whereas up to 50% of UC patients and 30% of CD patients have an indolent course of the disease.^[19–21]^

Among the clinical differences in univariate analysis, we found significant discrepancies for both diseases in the use of biological therapies, endoscopic activity, disease duration, sex, and extension/location of the disease. Regarding biological therapies in Chile, the government grants full coverage under the “Ricarte Soto” law for anti-TNFα therapy using a step-up approach, although in some cases with a severe phenotype, it cannot be immediately included in the funding program until the use of immunomodulatory treatment has failed after a 6-month course of therapy.^[22,23]^ The payment for other biological therapies or new small molecules is prohibited for most Chilean patients, and enrollment in clinical trials is sometimes the only chance to access therapies other than anti-TNFα. This Chilean healthcare stipulation would explain the difference in the application of biological therapies, and may be an area for further studies to compare IBD outcomes.

The influence of race on the choice of therapy and the biological response is an important issue to consider in further studies.^[24]^ One study showed that the non-white race is associated with decreased efficacy of anti-TNF.^[24]^ In addition, some studies have suggested a role for ethnicity and implicitly for genetic predisposition in modulating the risk of anti-TNF immunogenicity and treatment responsiveness.^[25]^ Recently, anti-TNF pharmacokinetic differences have been reported between CD and UC, showing that CD has a more favorable profile than UC, including high trough drug levels and low anti-drug levels. Pharmacokinetic factors related to drug responses may be associated with disease progression and ethnicity; thus, further studies addressing this question are needed.^[26]^

Underdiagnosis could explain the later age at diagnosis and shorter disease duration in Chilean CD patients. However, in that case, we would expect a more aggressive disease with a more prevalent structuring and penetrating phenotype, and more resection surgeries in the Chilean cohort as a result of a long disease evolution without treatment. In fact, our data show a more limited extension of disease at diagnosis for both diseases, despite the reduced use of biological therapies and the later age of CD onset in our Chilean cohort. Several studies^[27]^ have suggested that the natural history of IBD varies according to the age of onset, indicating different contributions of genetics, immune response, and environment to the IBD phenotype across ages, with genetics having a greater impact in the early stages. In contrast, the environmental impact was stronger in the late stages.^[27]^ It would be interesting to evaluate the weight of these factors in Hispanics with different ages of onset. It would be especially relevant to explore the factors in Chilean patients that could favor a less aggressive phenotype.

Regarding the sex differences for both subtypes, the proportion of women was significantly higher in the Chilean cohort. However, there was a predominance of men in the US group, which can be explained by the fact that one of the centers in the UCSD dataset corresponds to a veteran hospital, where the male population is higher than the female population. Therefore, sex may be an important factor to consider.

PCA separated North Americans from Chileans for each disease (UC and CD). In the North American and Chilean groups, the main variables in each first PCA component were related to therapy use (biological and immunomodulators), the extent of disease in UC cases, and disease location in CD patients. Because PCA is a simple linear method, which is usually used as a baseline,^[28]^ we confirmed the separation between the North American and Chilean groups for Crohn and UC using other ML methods. However, a few patients clustered with the opposite center (Fig. 3), which may be explained by the more diverse ethnicities in the US sample; 17% of CD patients and 28% of UC patients had Hispanic/Latin roots in our North American study sample. It would be interesting to evaluate and compare ancestry in future studies using larger sample sizes.

Surprisingly, although the univariate analysis did not show a difference in clinical activity scores (total Mayo Score for UC and Harvey Bradshaw Index) between the Chilean and North American cohorts, supervised clustering revealed clusters among Chileans with a higher clinical score.

Our RF models were also highly accurate in predicting the cohort for both UC and CD, although other methods, such as MLP and AdaBoost, also achieved similar results (data shown in Table 3, Figure S4, Supplemental Digital Content, http://links.lww.com/MD/H85, and Table S4, Supplemental Digital Content, http://links.lww.com/MD/H86). The Gini index confirmed that the main variables predicting UC nationality were related to the use of biological therapies, aminosalicylates, inflammatory parameters, disease duration, and disease extension. For CD, duration, variables related to the use of therapies, age, disease duration, inflammatory parameters, clinical and endoscopic scores, and disease localization were the primary variables for predicting the cohort (Chilean vs North American). Furthermore, although class separation was visible in the PCA projections, it was difficult to measure whether this separation was correct, as this type of metric is not as robust as the supervised methods. However, the concordance of the results between the univariate, unsupervised, and supervised methods was sufficiently robust to conclude that there were 2 distinct populations with the same disease for both CD and UC, mainly corresponding to different countries.

Differences in the location of disease at diagnosis were significant in the present study. In CD, inflammation can occur anywhere in the gastrointestinal tract, whereas UC is confined to the colon.^[29,30]^ The universally adopted Montreal classification distinguishes sub-phenotypes in UC by disease extent and age of onset, and in CD by disease location, behavior, and age of onset.^[29,30]^ In the CD Chilean cohort, colonic disease (L2) was more prevalent, whereas in North American patients, ileocolonic (L3) disease was most often observed. Proctitis and left-sided colitis were observed more frequently in Chilean than in North American patients. However, it should be noted that the extent of the disease was unknown in an important percentage of the data from the UC North American sample. Previous studies on CD that compared disease localization among patients from different ethnic groups found a higher proportion of white patients with isolated ileal disease than African Americans, Hispanics, or Asians.^[4,31,32]^ In our study, isolated ileal disease in Chilean patients was 11.6% compared to 9% in the North American population. Additionally, multiple UC studies found no significant differences in disease location between Hispanic and Caucasian patients, whereas one study found an increased frequency of extensive colitis in Hispanic patients compared with Caucasian patients.^[4,10,32,33]^ It should also be noted that all Hispanics have traditionally been bracketed within the same ethnic group, but differences between Hispanics from Spain and those from Latin America in immune disorders such as systemic lupus erythematosus have been reported.^[34]^

Interestingly, the differences in disease localization found in our study may reflect dissimilarities among populations at the genetic, microbial, and immunological levels. In terms of genetics, 3 loci (nucleotide-binding oligomerization domain-containing protein 2, major histocompatibility complex, and macrophage-stimulating protein-1 3p21) have been associated with IBD subphenotypes, particularly with disease location.^[35]^ For example, nucleotide-binding oligomerization domain-containing protein 2 variants have been related to small-intestinal CD, similarly as IL-23R and HLA-DRB1*01:03 variants have been related to colonic CD.^[35,36]^ The IBD Genetic Consortium conducted the most extensive genotype-phenotype association study involving 29,838 IBD patients from Europe, North America, and Australia.^[35]^ This group generated a risk score to test the hypothesis that colonic CD, ileal CD, and UC can be genetically separated.^[35]^ They found ileal and ileocolonic CD risk scores were closer and distinct from colonic CD. In contrast, colonic CD had an intermediate risk score between that of ileal/ileocolonic CD and UC. These findings support the concept that IBD should be classified into 3 groups: ileal CD, colonic CD, and UC.^[35]^ This finding also supports the idea that disease location is an intrinsic aspect of a patient’s disease, in part genetically determined, and could be a driver to changes in disease behavior over time.^[35]^ This concept is relevant considering that the Montreal classification stratified by location (L1: terminal ileum, L2: colon, L3: ileocolonic, and L4: upper GI location) evolved from an effort to stratify patients at higher risk for disease progression, identifying those who would benefit from an early combination therapy with immunosuppressants and biologics.^[37]^ Nevertheless, although disease location after diagnosis remains stable over time, differences exist in presentation and risk for progression or complications based on location, influencing clinical management.^[37]^ For example, Ileal CD is more often associated with the development of a fistulizing phenotype compared with isolated colonic disease.^[38]^ Moreover, post hoc analysis of the REACT trial showed that ileocolonic and isolated ileal disease was associated with an increased risk of disease-related complications or surgery.^[39]^ Whereas, when the compromise is colonic, extraintestinal manifestations are more likely.^[39,40]^ As for drug response, certolizumab and ustekinumab exhibit less effectiveness in ileal-CD, whereas vedolizumab induces less endoscopic improvement in ileal disease, and adalimumab has higher mucosal healing in colonic CD.^[40–43]^ Finally, it would be of great interest to evaluate the influence of ethnicity in further IBD-therapy studies according to the genetic risk score that include populations such as Latin American patients.

Differences in the location of the disease may also indicate variations in immune response. There is a regional variation in the relative abundance of immune cells along the healthy colon.^[44]^ Indeed, increased CD8^+^ T-cell resident memory, CD19^+^ B cells, and monocytes in the ascending colon have been reported.^[44]^ On the other hand, the transverse colon displays an increased abundance natural killer and γδ T cells, and the left colon and rectum have enrichment for antibody-secreting cells and CD4^+^ T cells.^[44]^ Integrins are cell-adhesion receptors expressed on leukocytes and play pivotal roles in IBD, being major mediators of the dysregulated traffic of lymphocytes and other immune cells to the inflamed intestine. Integrin expression varies throughout the colon; αE integrin expression is increased in ileum relative to colon, being unaffected by disease activity or therapy use.^[45]^ αEβ7+ T cells are highest in the ileum, with a decreasing gradient along the intestine and colon.^[45]^ T lymphocytes expressing α4β7 are lower in the ileum and higher in the descending colon, whereas frequencies of dendritic cells expressing αEβ7 are lower in the ascending colon than in the descending colon, with unknown expression in the ileum.^[45]^ Furthermore, there is segmental epithelial cell heterogeneity; Paneth cells that are highly specialized secretory cells that produce antimicrobial peptides are predominant in the small intestine, whereas this type of cell is scarce in the ascending colon.^[46]^ Another anatomical difference is the presence of the Peyer patches that are exclusive to the small intestine and mostly found in the terminal ileum.^[47]^ Peyer patches might induce immune tolerance or defense against pathogens resulting from the complex interplay between immune cells located in the lymphoid follicles and the follicle-associated epithelium.^[47]^ Both Paneth cells and Peyer patches have a role in IBD.^[46,47]^

Variations in the immune response have also been observed in the mesenteric tissue. Creeping fat (CF) corresponds to hyperplasia of mesenteric fat tissue that wraps around inflamed gut segments. Previous studies have demonstrated that bacterial translocation to the mesenteric fat induces immune infiltration in CF.^[48]^ One study has shown that CF in the small intestine is characterized by adipocyte hyperplasia, fibrosis, and intense infiltration of immune cells.^[49]^ Conversely, mesenteric fat adjacent to the colon lacked these findings.^[49]^ T-cell populations differed between mesenteric fat in ileal CD, colonic CD, and UC: the proportions of regulatory and central memory T cells were significantly higher in ileal CF than colonic fat in CD and UC. The ileal CF has nearly 10 times more T cells than colonic fat.^[49]^ Moreover, anatomic variation in the mesenteric macrophage phenotype has been described.^[50]^

The abundance and diversity of the microbiome throughout the entire gut increase from the proximal to the distal intestine and are influenced by microbial community dynamics and host features. Some bacteria are related to the diseased segment; adherent-invasive *Escherichia coli* has been found in the ileum of patients with CD.^[51,52]^ Focusing on the site, rectal and ileal samples have reduced Firmicutes abundance (*Faecalibacterium prausnitzii*) and increased Proteobacteria (*E coli*).^[53]^ The *F prausnitzzi*/*E coli* ratio might discriminate between healthy individuals and those with ileal, ileocolonic, or isolated colonic CD.^[54]^ These findings support those site-specific changes in microbiota might be associated with the subclassification of IBD based on localization.^[40]^ Another critical aspect to consider is that geographic location is a significant determinant of microbiota variation in IBD, and ethnicity, diet, and geographical locations need to be considered in future microbiota studies with implications for the prospect of personalized therapeutics.^[55]^

A bias of this study was the limited sample size of 265 patients. Many AI algorithms exhibit improved accuracy when used on large amounts of data; deep neural networks and Bayesian nonparametric models are the best examples of such phenomena. To take advantage of this improved accuracy, AI research is often conducted with large training sets allowing performance to be evaluated based on a great deal of data. Our aim has not been to develop general-purpose AI algorithms, but to compare 2 populations of different racial features and the impacts those features have on their IBD outcomes. In this sense, the challenge of our study has been that the amount of data is fixed and small, and thus we were unable to take advantage of the known property of AI algorithms that work best for large datasets. To deal with this challenge, we combined supervised and unsupervised methods that can operate in the “small data regime.” For instance, one of the techniques we used was Random Forest, whose success has been validated even in the small-data/high-dimensional case (aka small N, large P)^[56]^ and for other clinical applications.^[57,58]^

Furthermore, additional ML methods corroborated and revealed that Chilean Latin American and North American groups exhibited different clinical patterns (Table S4, Supplemental Digital Content, http://links.lww.com/MD/H86). These results encouraged us to pursue further studies by using more extensive databases.

## 5. Conclusion

Although this study was conducted on a relatively small dataset, our results suggest clinical differences between these 2 population groups: a later CD age at diagnosis with a predominantly less aggressive phenotype (39% vs 54% B1) and a more localized disease despite fewer biological therapies used in Chile for both CD and UC.

Our study showed that ML tools can help to characterize and distinguish IBD populations, unraveling novel associations between clinical variables and specific population characteristics. This approach can lead to improved disease stratification, which may lead to essential discoveries in clinical outcomes, new genetic polymorphisms, and differences in the microbiota that may lead to differences in treatment and prognosis, among others. This highlights the usefulness of additional translational studies to compare the influence of ethnicity on IBD outcomes in a wide range of other immune-mediated diseases.

## Author contributions

TPJ, BP, FT, MO, MCV, and MAL for study concept and design. TPJ, BP, and FT drafted the manuscript. TPJ, ADV, MC, BC, VS, EA, RE, and JRN for data acquisition. TPJ, BP, GA, MO, DA, MCV, and FT for data analysis and interpretation. TPJ, BP, MO, DA, and PB for statistical analyses. MAL, GA, CH, MCV, and FT for critical revision of the manuscript.

**Conceptualization:** Tamara Pérez-Jeldres, Benjamín Pizarro, Mauricio Cerda-Villablanca, Felipe Tobar.

**Data curation:** Tamara Pérez-Jeldres, Benjamín Pizarro, Danilo Alvares, Andrés de la Vega, Bárbara Cornejo, Verónica Silva, Elizabeth Arriagada, Jesús Rivera-Nieves, Ricardo Estela, Macarena Cannistra.

**Formal analysis:** Tamara Pérez-Jeldres, Benjamín Pizarro, Gabriel Ascui, Matías Orellana, Danilo Alvares, Pablo Baéz, Felipe Tobar.

**Funding acquisition:** Tamara Pérez-Jeldres.

**Investigation:** Tamara Pérez-Jeldres, Benjamín Pizarro, Matías Orellana, Felipe Tobar.

**Methodology:** Tamara Pérez-Jeldres, Benjamín Pizarro, Danilo Alvares, Manuel Álvarez Lobos, Felipe Tobar.

**Project administration:** Tamara Pérez-Jeldres, Benjamín Pizarro, Felipe Tobar.

**Resources:** Andrés de la Vega.

**Software:** Benjamín Pizarro, Danilo Alvares.

**Supervision:** Tamara Pérez-Jeldres.

**Validation:** Benjamín Pizarro, Gabriel Ascui.

**Visualization:** Benjamín Pizarro, Gabriel Ascui.

**Writing – original draft:** Tamara Pérez-Jeldres, Benjamín Pizarro.

**Writing – review & editing:** Tamara Pérez-Jeldres, Gabriel Ascui, Mauricio Cerda-Villablanca, Cristián Hernández-Rocha, Manuel Álvarez Lobos, Felipe Tobar.

## Supplementary Material


