# Supplementary material for: Ethnicity influences phenotype and clinical outcomes: Comparing a South American with a North American inflammatory bowel disease cohort
Source: Medicine (Baltimore). 2022 Sep 9;101(36):e30216. doi: 10.1097/MD.0000000000030216 (PMC10980497; doi:10.1097/MD.0000000000030216)
Supplement: Supplementary file 1 [file medi-101-e30216b-s001.pdf]

Supplemental Digital Content. Table S1. Relationship between clinical parameters and extension (UC) disease in North American and Chilean IBD patients. a) Continuous variables (Mann-Whitney U test); b) Categorical variables (Fisher's exact test).

a)

Mann-Whitney U tests	Disease Location		
**Variable**	**E1**	**E2**	**E3**
**Age at Diagnosis**	-	0.315	0.508
**White Cells**	-	0.800	0.591
**Plateles**	-	0.768	0.079
**Albumin**	-	1.000	0.026
**Hemoglobin**	-	0.015	0.511
**CRP**	-	0.269	0.488
**Duration of Disease**	-	1.000	0.082

b)

Fisher's exact tests	Disease Location		
**Variable**	**E1**	**E2**	**E3**
**Family history of IBD**	-	1.000	1.000
**Sex**	-	0.007	0.134
**Hospitalization for IBD**	-	0.108	0.809
**Smoking**	-	1.000	0.048
**Prior history of esteroids use**	-	0.183	0.644
**Prior history of immunomodulators use**	-	0.628	0.041
**Prior history of biological therapy use**	-	0.093	0.000
**History of discontinuation or anti-TNF failure**	-	0.129	0.000
**Naive anti-TNF**	-	0.007	0.000
**Current esteroids therapy**	-	1.000	0.107
**Current biological therapy**	-	0.086	0.000
**Current anti-TNF therapy**	-	0.268	0.008
**Current immunomodulator use**	-	0.384	0.386

Supplemental Digital Content. Table S2. Relationship between clinical parameters and location (CD) disease in North American and Chilean IBD patients. a) Continuous variables (Mann-Whitney U test); b) Categorical variables (Fisher's exact test).

a)

Mann-Whitney U tests	**Disease Location**		
**Variable**	**L1**	**L2**	**L3**
**Age at Diagnosis**	0.115	0.023	0.782
**White Cells**	1.000	0.208	0.213
**Plateles**	0.706	0.658	0.275
**Albumin**	0.495	0.677	0.005
**Hemoglobin**	0.261	0.246	0.207
**CRP**	0.152	0.482	0.480
**Duration Disease**	1.000	0.124	0.890

b)

Fisher's exact tests	**Disease Location**		
**Variable**	**L1**	**L2**	**L3**
**Family history of IBD**	1.000	0.072	0.335
**Sex**	1.000	0.099	0.721
**Hospitalization for IBD**	1.000	1.000	0.086
**Smoking**	1.000	1.000	0.651
**Prior history of esteroids use**	1.000	0.556	0.601
**Prior history of immunomodulators use**	0.447	0.281	0.688
**Prior history of biological therapy use**	0.079	0.006	0.004
**History of discontinuation or anti-TNF failure**	0.284	0.002	0.037
**Naive anti-TNF**	0.111	0.012	0.001
**Current esteroids therapy**	1.000	0.563	0.398
**Current biological therapy**	0.053	0.006	0.005
**Current anti-TNF therapy**	0.032	0.223	0.157
**Current immunomodulator use**	0.521	0.589	0.732

Supplemental Digital Content. Table S3. Proportion of patients with Crohn´s disease undergoing biological therapies according to localization and center.

**L1 (Ileal)**	**Current biological therapy**		**p-Value**
Center	No	Yes	
USA	0	2	0.053
Chile	15	3	
**L2 (Colonic)**	**Current biological therapy**		**p-Value**
Center	No	Yes	
USA	0	4	0.006
Chile	27	8	
**L3 (ileocolonic)**	**Current biological therapy**		**p-Value**
Center	No	Yes	
USA	3	14	0.005
Chile	11	5	
