# Supplementary figures and images for: Ethnicity influences phenotype and clinical outcomes: Comparing a South American with a North American inflammatory bowel disease cohort
Source: Medicine (Baltimore). 2022 Sep 9;101(36):e30216. doi: 10.1097/MD.0000000000030216 (PMC10980497; doi:10.1097/MD.0000000000030216)

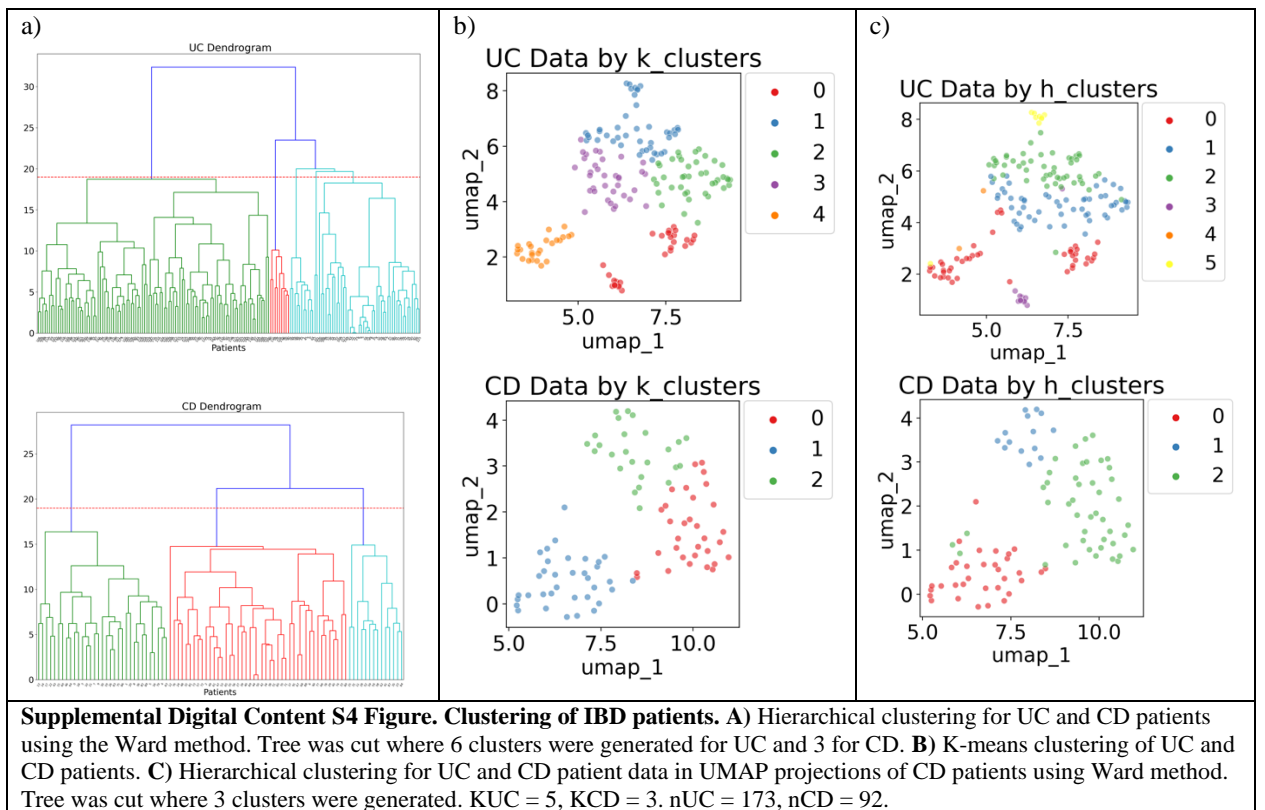

Supplement: Supplementary file 2 [file medi-101-e30216b-s002.pdf]

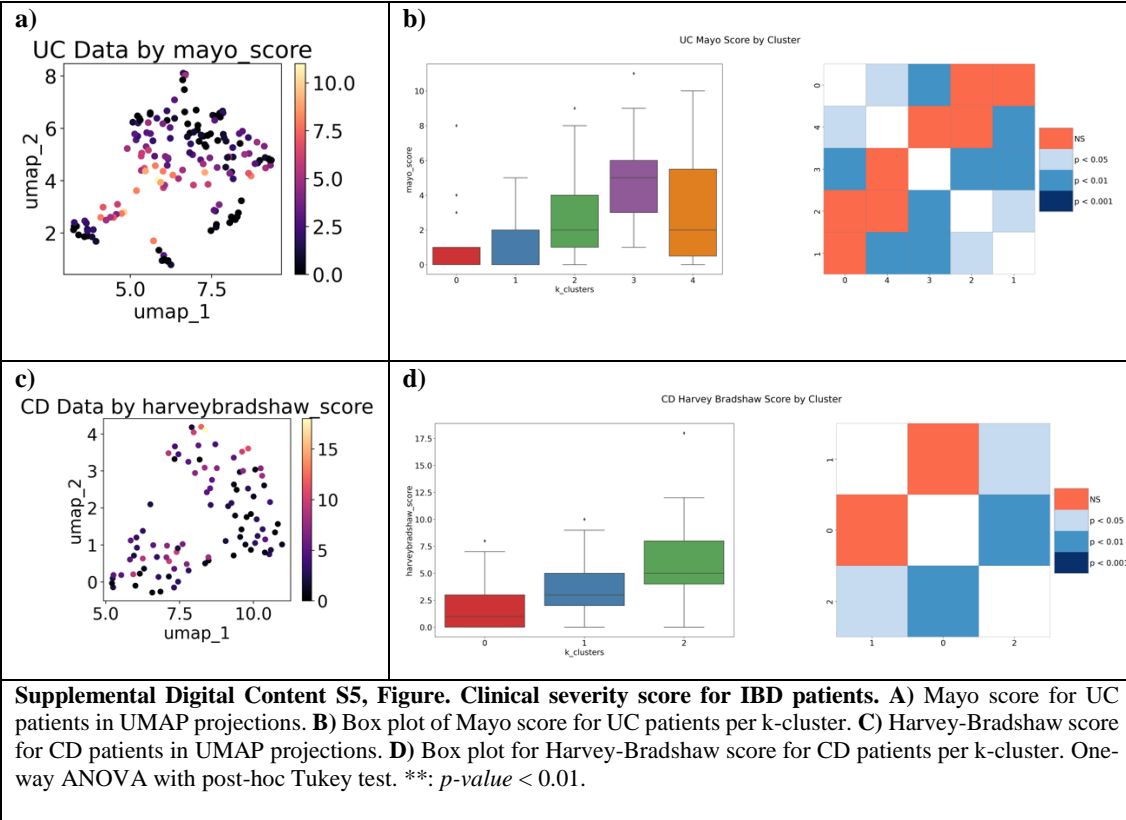

Supplement: Supplementary file 3 [file medi-101-e30216b-s003.pdf]

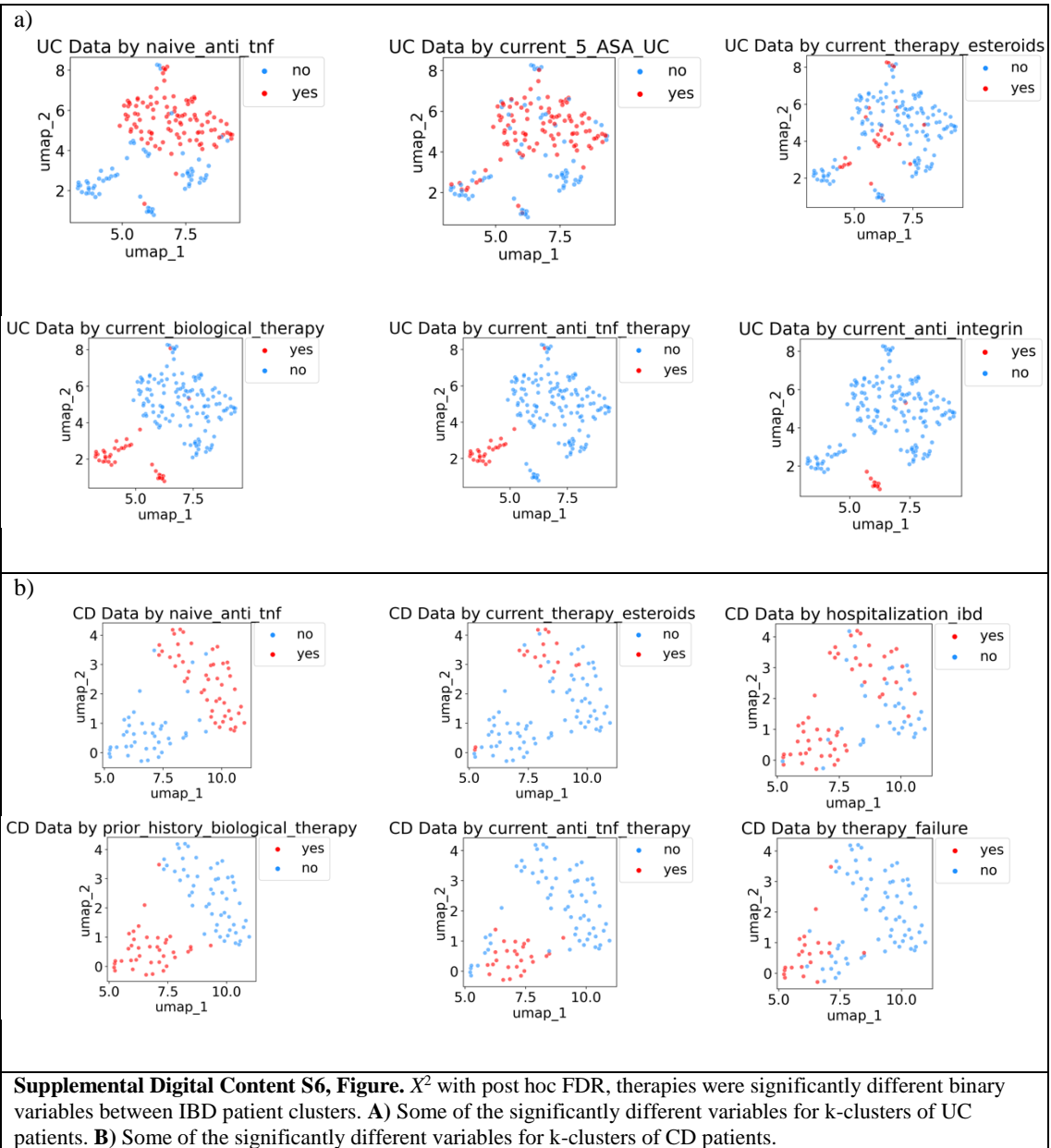

Supplement: Supplementary file 4 [file medi-101-e30216b-s004.pdf]
