# Supplementary material for: Ethnicity influences phenotype and clinical outcomes: Comparing a South American with a North American inflammatory bowel disease cohort
Source: Medicine (Baltimore). 2022 Sep 9;101(36):e30216. doi: 10.1097/MD.0000000000030216 (PMC10980497; doi:10.1097/MD.0000000000030216)
Supplement: Supplementary file 5 [file medi-101-e30216b-s005.pdf]

a) b)

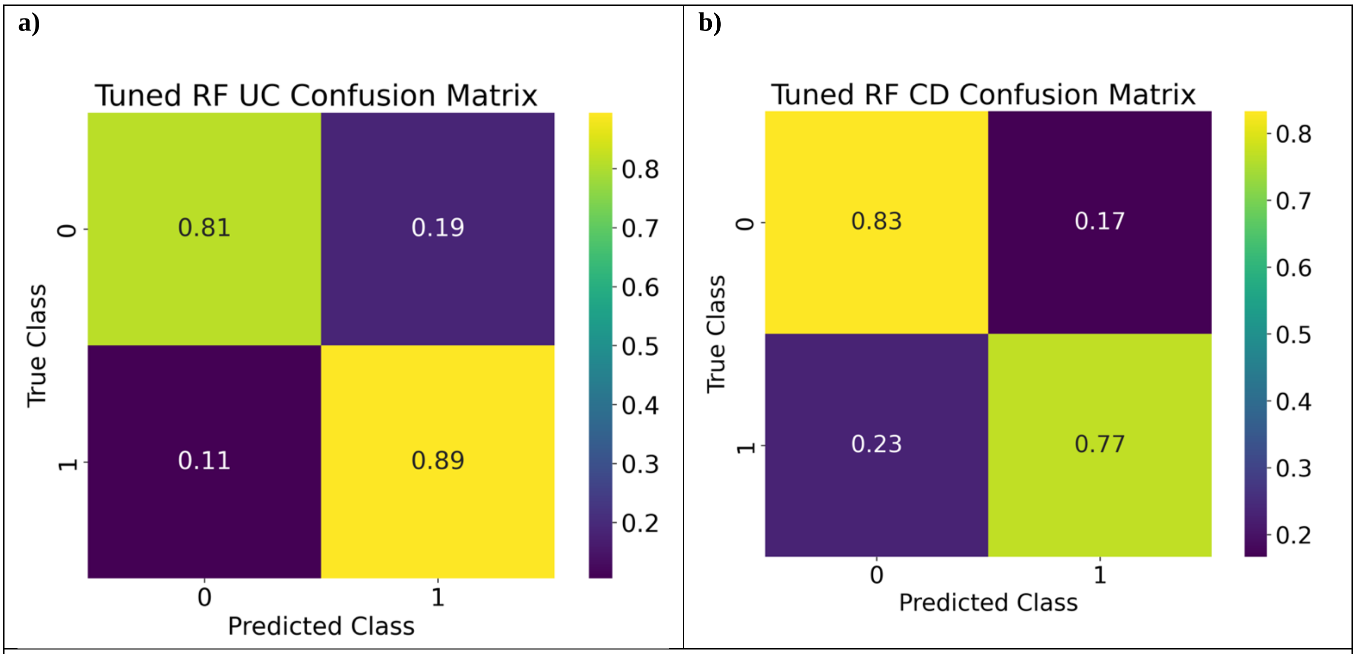

Supplemental Digital Content S7, Figure. Confusion Matrix for cohort prediction. In the X-axis, the confusion matrix shows the true class, i.e., the center to which the patient belongs, while in the Y-axis, it shows the predicted class of the RF model using the clinical data of the patients. A perfect classification will lead to a diagonal full of ones. a) UC model correctly classifies 81% of US patients and 89% of Chilean patients. b) CD model correctly classifies 83% of US patients and 77% of Chilean patients. 0: US patients, 1: Chilean.
