# Supplementary material for: Ethnicity influences phenotype and clinical outcomes: Comparing a South American with a North American inflammatory bowel disease cohort
Source: Medicine (Baltimore). 2022 Sep 9;101(36):e30216. doi: 10.1097/MD.0000000000030216 (PMC10980497; doi:10.1097/MD.0000000000030216)
Supplement: Supplementary file 6 [file medi-101-e30216b-s006.pdf]

Supplemental Digital Content S8, Table. Comparison of different supervised machine learning algorithms for center prediction in) Ulcerative Colitis patients. b) Crohn´s disease patients.

a)

UC Center Prediction			
Model	Precision	Recall	f1-score
Support -Vector Machine	0.71	0.71	0.71
Logistic Regression	0.74	0.74	0.74
AdaBoost	0.86	0.86	0.86
Multi Layer Perceptron	0.87	0.86	0.85
Linear Discriminant Analysis	0.81	0.80	0.80
Random Forest	0.86	0.86	0.86
For Precision. Recall and f1-Score weighted average was used			

b)

Crohn´s Disease Center Prediction			
Model	Precision	Recall	f1-score
Support-Vector Machine	0.69	0.63	0.64
Logistic Regression	0.69	0.63	0.64
AdaBoost	0.82	0.79	0.80
Multi Layer Perceptron	0.69	0.63	0.64
Linear Discriminant Analysis	0.50	0.53	0.51
Random Forest	0.82	0.79	0.80
For Precision. Recall and f1-Score weighted average was used			
